# Electrical stimulation to regain lower extremity muscle perfusion and endurance in patients with post‐acute sequelae of SARS CoV‐2: A randomized controlled trial

**DOI:** 10.14814/phy2.15636

**Published:** 2023-03-10

**Authors:** Alejandro Zulbaran‐Rojas, Myeounggon Lee, Rasha O. Bara, Areli Flores‐Camargo, Gil Spitz, M. G. Finco, Amir Behzad Bagheri, Dipaben Modi, Fidaa Shaib, Bijan Najafi

**Affiliations:** ^1^ Interdisciplinary Consortium on Advanced Motion Performance (iCAMP), Division of Vascular Surgery and Endovascular Therapy, Michael E. DeBakey Department of Surgery Baylor College of Medicine Houston Texas USA; ^2^ Baylor St Luke's Medical Center, Exercise Physiology Liver Transplant Program Houston Texas USA; ^3^ Department of Pulmonary Critical Care Baylor College of Medicine Houston Texas USA

## Abstract

Muscle deconditioning and impaired vascular function in the lower extremities (LE) are among the long‐term symptoms experienced by COVID‐19 patients with a history of severe illness. These symptoms are part of the post‐acute sequelae of Sars‐CoV‐2 (PASC) and currently lack evidence‐based treatment. To investigate the efficacy of lower extremity electrical stimulation (E‐Stim) in addressing PASC‐related muscle deconditioning, we conducted a double‐blinded randomized controlled trial. Eighteen (*n* = 18) patients with LE muscle deconditioning were randomly assigned to either the intervention (IG) or the control (CG) group, resulting in 36 LE being assessed. Both groups received daily 1 h E‐Stim on both gastrocnemius muscles for 4 weeks, with the device functional in the IG and nonfunctional in the CG. Changes in plantar oxyhemoglobin (OxyHb) and gastrocnemius muscle endurance (GNMe) in response to 4 weeks of daily 1 h E‐Stim were assessed. At each study visit, outcomes were measured at onset (*t*_0_), 60 min (*t*_60_), and 10 min after E‐Stim therapy (*t*_70_) by recording ΔOxyHb with near‐infrared spectroscopy. ΔGNMe was measured with surface electromyography at two time intervals: 0–5 min (Intv_1_) and: 55–60 min (Intv_2_). Baseline OxyHb decreased in both groups at *t*_60_ (IG: *p* = 0.046; CG: *p* = 0.026) and *t*_70_ (IG = *p* = 0.021; CG: *p* = 0.060) from *t*_0_. At 4 weeks, the IG's OxyHb increased from *t*_60_ to *t*_70_ (*p* < 0.001), while the CG's decreased (*p* = 0.003). The IG had higher ΔOxyHb values than the CG at *t*_70_ (*p* = 0.004). Baseline GNMe did not increase in either group from Intv_1_ to Intv_2_. At 4 weeks, the IG's GNMe increased (*p* = 0.031), whereas the CG did not change. There was a significant association between ΔOxyHb and ΔGNMe (*r* = 0.628, *p* = 0.003) at 4 weeks in the IG. In conclusion, E‐Stim can improve muscle perfusion and muscle endurance in individuals with PASC experiencing LE muscle deconditioning.

## Graphical abstract

This study indicates that self‐administered lower extremity (LE) electrical stimulation (E‐Stim) therapy is practical and effective at promoting the restoration of LE muscle perfusion and endurance in individuals with post‐acute sequelae of Sars‐CoV‐2 (PASC) who were previously hospitalized. The application of LE E‐Stim for 1 h daily over a 4 week period resulted in a significant increase in gastrocnemius muscle oxyhemoglobin levels, which led to an improvement in muscle endurance and recovery of excess postexercise oxygen consumption

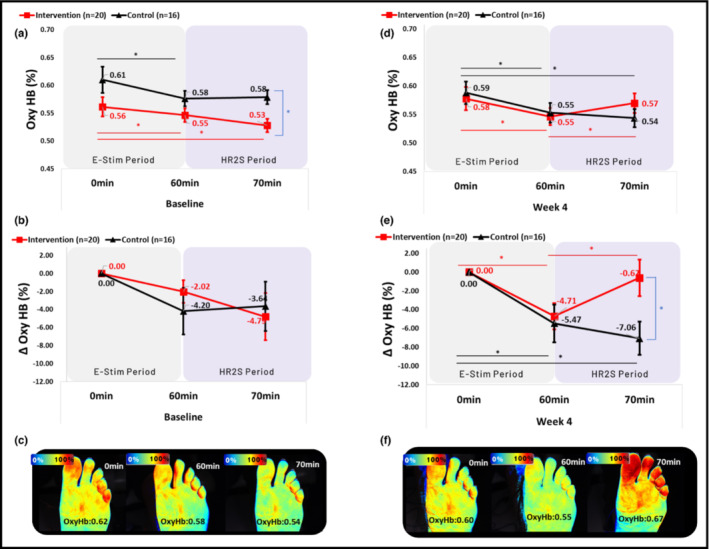

## 1 INTRODUCTION

The novel coronavirus disease 2019 (COVID‐19) pandemic has generated great illness, death, distress, and undefined sequelae on our society (Cucinotta & Vanelli, 2020). With vaccines and monoclonal therapies, moderate to severe cases of COVID‐19 infection diminished markedly (Hwang et al., 2022; Peng et al., 2021). However, COVID‐19 survivors that were inflicted with severe acute illness, in particular those who required prolonged bed rest, still suffer from post‐acute sequelae of Sars‐CoV‐2 (PASC) (Parker et al., 2021).

According to Centers for Disease Control and Prevention (Centers for Disease Control and Prevention, 2022), PASC can persist for up to 2 years after recovery (Huang et al., 2022), and even a mild course of acute infection may lead to long‐term disability (Taquet et al., 2021). Musculoskeletal sequelae are a key concern for clinicians treating PASC (Disser et al., 2020), as they can lead to debilitating outcomes for survivors who were hospitalized or immobilized for extended periods (de Andrade‐Junior et al., 2021; Nalbandian et al., 2021). Musculoskeletal sequelae are characterized by atrophy, weakness, pain, and fatigue, and since these issues are often located in the lower extremities (LE) (Heesakkers et al., 2022; Parry & Puthucheary, 2015), they can significantly impact daily activities. In particular, LE muscle weakness has been associated with reduced functional abilities in individuals with PASC (Shanbehzadeh et al., 2021).

The mechanism by which Sars‐CoV‐2 damages muscles is not yet fully understood. There is speculation that microcirculation deterioration may be a factor (Trinity et al., 2021), possibly resulting from vascular endothelial damage through ACE2 receptors (Amraei & Rahimi, 2020) or a viral‐induced hyper‐inflammatory state that can cause myofibrillar breakdown, mitochondrial dysfunction, and muscle degradation (Piotrowicz et al., 2021). Other studies suggest that during severe acute COVID‐19 infection, hyperlactemic states can lead to a deoxygenation of the musculoskeletal system, impairing the transportation of oxygen to distal tissues, and resulting in hypoxia/ischemia (Seixas et al., 2022). As a result, COVID‐19 patients who experienced severe illness have been shown to have lower vascular function and blood flow velocity, vessel inflammation, and arterial stiffness in the LE (Disser et al., 2020; Paneroni et al., 2021; Ratchford et al., 2021).

Physical therapy programs have been proposed for the management of musculoskeletal PASC (Righetti et al., 2020). However, they may not adequately address the vascular impairment induced by COVID‐19. Recent evidence suggests that individuals with PASC may experience marked hypoxia (Fuglebjerg et al., 2020; Singh et al., 2022) and a poor hemodynamic response to stress (HR2S) in the LE, which can affect exercise tolerance (Serviente et al., 2022). Waiting to engage in mobility programs may be detrimental (O'Sullivan et al., 2021), so safe, and effective solutions to improve HR2S are needed to support functional recovery in this population.

A number of studies support the effectiveness of electrical stimulation (E‐Stim) to improve LE vascular health (Gorgey et al., 2009; Hamid & Hayek, 2008; Li et al., 2017). E‐Stim involves the delivery of preprogrammed trains of stimuli to superficial muscles via adhesive pads, which can evoke submaximal muscle contractions by recruiting motor units in a nonselective, spatially fixed, and temporally synchronous pattern (Maffiuletti et al., 2019). This therapy has been shown to improve muscle endurance in hospitalized or limited‐mobility patients (Burgess et al., 2021), to reduce muscle loss (Burgess et al., 2021; Leite et al., 2018), and improve tissue perfusion (Zulbaran‐Rojas et al., 2021). Additionally, E‐Stim has been effective in improving muscle strength (Righetti et al., 2022) and endurance (Zulbaran‐Rojas et al., 2022) in severe acute COVID‐19 patients. However, the long‐term effects of E‐Stim on muscle perfusion have not been well studied, and its utility for the recovery of individuals with musculoskeletal PASC has not been explored.

Given the poor exercise tolerance and potential for unhealthy HR2S in individuals with PASC, safe and effective solutions for improving HR2S and supporting functional recovery are needed. Therefore, the purpose of our study was to investigate the potential benefits of E‐Stim in improving the recovery of individuals with musculoskeletal PASC. Our main hypothesis is that E‐Stim therapy will improve both HR2S and LE muscle endurance in this population. Moreover, we hypothesize that there will be a positive correlation between muscle perfusion and endurance, indicating that E‐Stim may improve both aspects of muscle function in individuals with musculoskeletal PASC.

## 2 METHODS

### 2.1 Study population

A double‐blinded randomized controlled trial of individuals experiencing persistent LE musculoskeletal PASC was conducted. Participants were recruited from the Baylor College of Medicine (BCM) Post‐COVID‐19 Care Clinic (Houston, TX, USA) between November 2021 and May 2022. All participants signed an informed consent approved by the local Institutional Review Board (IRB #H‐47781) before study enrollment. The study was registered on ClinicalTrials.gov (Identifier: NCT05198466) and followed the Consolidated Standards of Reporting Trials (CONSORT) guidelines for randomized clinical trials.

Participants were included if they were previously hospitalized due to acute COVID‐19 infection, aged 18–85 years old, diagnosed with PASC by a pulmonologist and critical care clinician (F.S., D.M), and reported persistent LE musculoskeletal symptoms such as atrophy, weakness, numbness, and/or pain at their first consultation. Those who had demand‐type cardiac pacemaker, implanted defibrillator, active wound infection, or below the knee amputation were excluded.

Demographic and clinical characteristics were recorded from the electronic medical records. Other baseline assessments included depression by the Center for Epidemiologic Studies Depression Scale (CES‐D) (Weissman et al., 1977), cognition by the Montreal Cognitive Assessment (MoCA) (Nasreddine et al., 2005), anxiety by the Beck Anxiety Inventory Scale (BAI) (Beck et al., 1988), pain by the visual‐analog‐scale (VAS) (Langley & Sheppeard, 1985), quality‐of‐life by the Patient‐Reported Outcomes Measurement Information System (PROMIS) (Cella et al., 2010), sleep quality by Pittsburgh‐Sleep‐Quality‐Index (PSQI) (Buysse et al., 1989), and activity of daily living by the Katz Index and Lawton scale (Katz, 1983; Lawton & Brody, 1969).

### 2.2 Randomization, group allocation, and intervention

Participants were randomized (ratio: 1:1) to either control (CG) or intervention (IG) groups through a computer‐generated list followed by sequential allocation. Participants and care providers were blinded to the group allocation. Investigators who collected and analyzed the data were not blinded. The IG received E‐Stim to the gastrocnemius muscle (GNM) via four electrode adhesive pads (Avazzia Inc), two placed on each leg. One pad was placed on the proximal GNM (Silva et al., 2017) while the other was placed on the Achilles tendon. A four‐pin lead wire was used to connect the E‐Stim device (Tenant Biomodulator®) to the electrode pads in both legs simultaneously. The CG was provided with an identical, but nonfunctional device (sham). Participants were instructed to self‐manage daily 1 h E‐Stim therapy at a time of their convenience to both LE for a course of 4 weeks. Weekly support phone calls by research assistants (A.Z., R.B., A.F.) were performed to monitor adherence. There were no lifestyle or dietary restrictions needed to apply E‐Stim during the study period.

E‐stim application was delivered by an interactive high voltage pulsed alternative current (HVPAC) in the shape of an asymmetrical damped sinusoidal biphasic pulsed waveform (Senergy Medical Group, n.d.), which allows muscle relaxation and avoids fatigue during therapy (Zulbaran‐Rojas et al., 2021). E‐Stim pulse duration was set between 400 and 1400 microseconds (μs), with a pulse frequency between 20 and 121 hertz (Hz). The E‐Stim sham device did not elicit electrical currents.

### 2.3 Procedures and outcome measures

**FIGURE 1:**
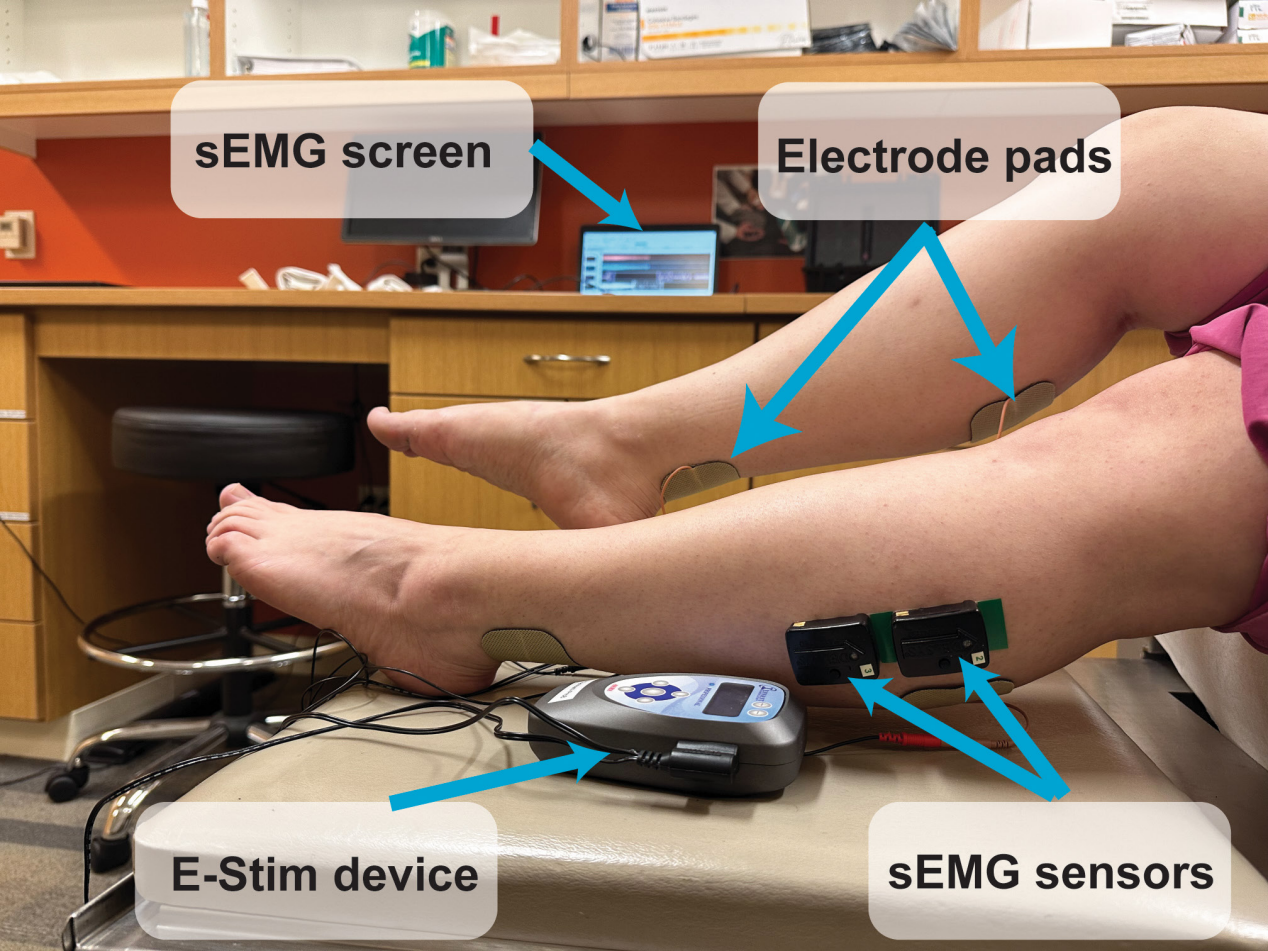
Study setup: electrical stimulation device, plugs and pads, and surface electromyography sensors. Participants received electrical stimulation through electrode adhesive pads placed on both proximal and distal gastrocnemius muscles using a bioelectric stimulation technology® (BEST) micro‐current platform (Tennant Biomodulator®). E‐Stim was active in the intervention group and nonfunctional in the control group. Two surface electromyography (Delsys Trino Wireless EMG System) sensors were placed on the proximal lateral gastrocnemius of each lower extremity to evaluate muscle endurance in response to E‐Stim. sEMG, surface electromyogram; E‐stim, electrical stimulation.

Outcomes were measured at the BCM Post‐COVID‐19 Care Clinic at baseline and 4 weeks visits during regular work hours (9:00 a.m. – 5:00 p.m.). Upon arrival to the hospital, participants were located on a regular exam chair in Fowler's position (60 degrees) with the legs extended (Figure 1). After resting for 5–10 min, approximate real‐time muscle‐perfusion was measured in response to 1 h E‐Stim therapy using a validated near‐infrared‐spectroscopy (NIRS) camera (Snapshot NIR, KENT Imaging Inc.). Oxyhemoglobin (OxyHb, defined as % of oxygenated hemoglobin) (Barstow, 1985) was obtained from the distal foot by tracing the metatarsal area including the five toes. From an exercise perspective, OxyHb allows for calculation of muscular efficiency/work executed by the muscle, the amount of oxygen consumption to produce a certain amount of work, and the velocity of muscle recovery after the work has ceased (Parker, 2021; Steinberg, 2022). When oxygen consumption is constant during steady‐state levels (i.e., isometric muscle contraction for 1 h), changes in NIRS signals should primarily reflect changes in oxygen delivery or uptake of a specific area (Fadel et al., 1985). However, when oxygen consumption surpasses the muscle supply during activity, the levels of OxyHb decrease (Beerthuizen, 1993). Under this concept, pictures were collected at three different time points within the baseline and 4‐weeks visits: (1) pre‐therapy, *t*_0_ (0 min) to record steady‐state basal levels, (2) end‐of‐therapy, *t*_60_ (60 min) to assess oxygen consumption (Dobson & Gladden, 1985), and (3) 10 min after stopping therapy, *t*_70_ to assess the reperfusion period (Meixner et al., 2022; Meneses et al., 2020) or HR2S.

During the 1 h E‐Stim session, changes in GNM endurance (GNMe, defined as sustained muscle involuntary contraction (Hagberg, 1981)) were recorded using surface electromyography (sEMG) at two time point intervals: (1) 0–5 min (Interval 1, Intv_1_), indicating therapy start; and (2) 55–60 min (Interval 2, Intv_2_), indicating end of therapy.

To evaluate GNMe, two sEMG sensors (Delsys Trino Wireless EMG System) were placed vertically next to each other at the lateral proximal GNM of each leg according to sEMG for a Non‐Invasive Assessment of Muscles (SENIAM) guidelines (Hermens et al., 2000). The sEMG data were collected at 2000 Hz and the raw sEMG signal was filtered using a fourth‐order Butterworth band‐pass filter with cutoff frequencies of 20 and 350 Hz from each sensor. Then, the sensor with less noise was used to quantify GNMe in response to E‐stim, the other sensor was discarded. Integrated EMG (iEMG) was calculated (Medved, 1999; Truong Quang Dang et al., 2012) to quantify the amount of muscle activation by motor units (Sleivert & Wenger, 1994). Then, iEMG was normalized by the average iEMG value extracted during the trial to compare the iEMG values in the baseline and 4 week visits (Allison et al., 1993; Morris et al., 1998).

### 2.4 Safety, feasibility, and acceptability

For patient safety, body saturation of oxygen (SatO_2_) was measured pre‐ and during therapy using a pulse oximeter (Santamedical Dual Color OLED) to monitor exercise‐induced (silent) hypoxia (Fuglebjerg et al., 2020; Rahman et al., 2021). Protocol delivery was set as ≥80%, accrual recruitment (≥2 patients/month), and ≥80% outcome measuring (Kho et al., 2019). Device acceptability was set as ≥80% assessed by ease of use questions based on a technology acceptance model questionnaire (Venkatesh & Davis, 2000). Moreover, adverse events throughout the study such as pain, skin damage, and discomfort were documented.

### 2.5 Sample size justification

Power analysis was conducted to calculate the minimum sample size using G*power software (version of 3.1.6) as follows: (1) Moderate effect size (Cohen's *d* = 0.5), (2) 80% power, (3) Alpha of 5%, (4) two number of groups, (5) three repeated measurements, and (6) 0.5 correlation among the repeated measurements. As a result, 28 samples were required. However, considering a dropout rate of up to 10%, a total of 32 samples were required to detect significance.

### 2.6 Statistical analysis

Each LE was considered as an independent sample due to the variability in muscular and vascular status (Häkkinen et al., 1997; Khan et al., 2019) as well as muscle strength asymmetry, dominance, and length discrepancy (Knutson, 2005; Laroche et al., 2012; Sadeghi et al., 2000). Shapiro–Wilk test was used to assess data normality (*p* > 0.05). Independent *t*‐tests, Chi‐square or Mann–Whitney U tests were used to compare baseline characteristics between groups. Effect size was measured using Cohen's d. Generalized Estimating Equations (GEE) was performed to assess the group*time interaction effect at baseline and 4 weeks represented by estimated means and standard errors. E‐Stim effect on GNMe (i.e., Intv_1_ and Intv_2_) and OxyHb (i.e., *t*_0_, *t*_60_, and, *t*_70_) were assessed within and between groups. Normalized GNMe and OxyHb values at each time point within the 1 h E‐Stim session were estimated having the first time point (i.e., *t*_0_ or Intv_1_) as 0% reference (i.e., [GNMe at Intv_1_ – GNMe at Intv_1_]/[GNMe at Intv_1_] * 100; [OxyHb at *t*_0_ − OxyHb at *t*_0_]/[OxyHb at *t*_0_] * 100) to all other time points (i.e., [GNMe at Intv_2_ − GNM_e_ at Intv_1_]/[GNMe at Intv_1_] * 100; [OxyHb at *t*_60_ or *t*_70_ − OxyHb at *t*_0_]/[OxyHb at *t*_0_] * 100). Results adjusted to potential confounders are included in the Supplementary Material. To compare the treatment effect at 4 weeks (i.e., active, sham), Delta (Δ) values of gastrocnemius muscle endurance GNMe (i.e., ΔGNMe = GNMe at Intrv_2_ − GNMe at Intrv_1_) and OxyHb (i.e., ΔOxyHb = OxyHb at *t*_70_ − OxyHb at *t*_0_) were calculated according to the E‐Stim duration (*t*_0_ *‐t*_60_) and additional reperfusion period (*t*_0_ *‐t*_70_), respectively. Pearson's correlation analysis was performed to explore the association between ΔGNMe and ΔOxyHb. All statistical analyses were performed using SPSS 28.0 (IBM), and the statistical significance level was set at *p* ≤ 0.05.

## 3 RESULTS

### 3.1 Clinical characteristics

**FIGURE 2:**
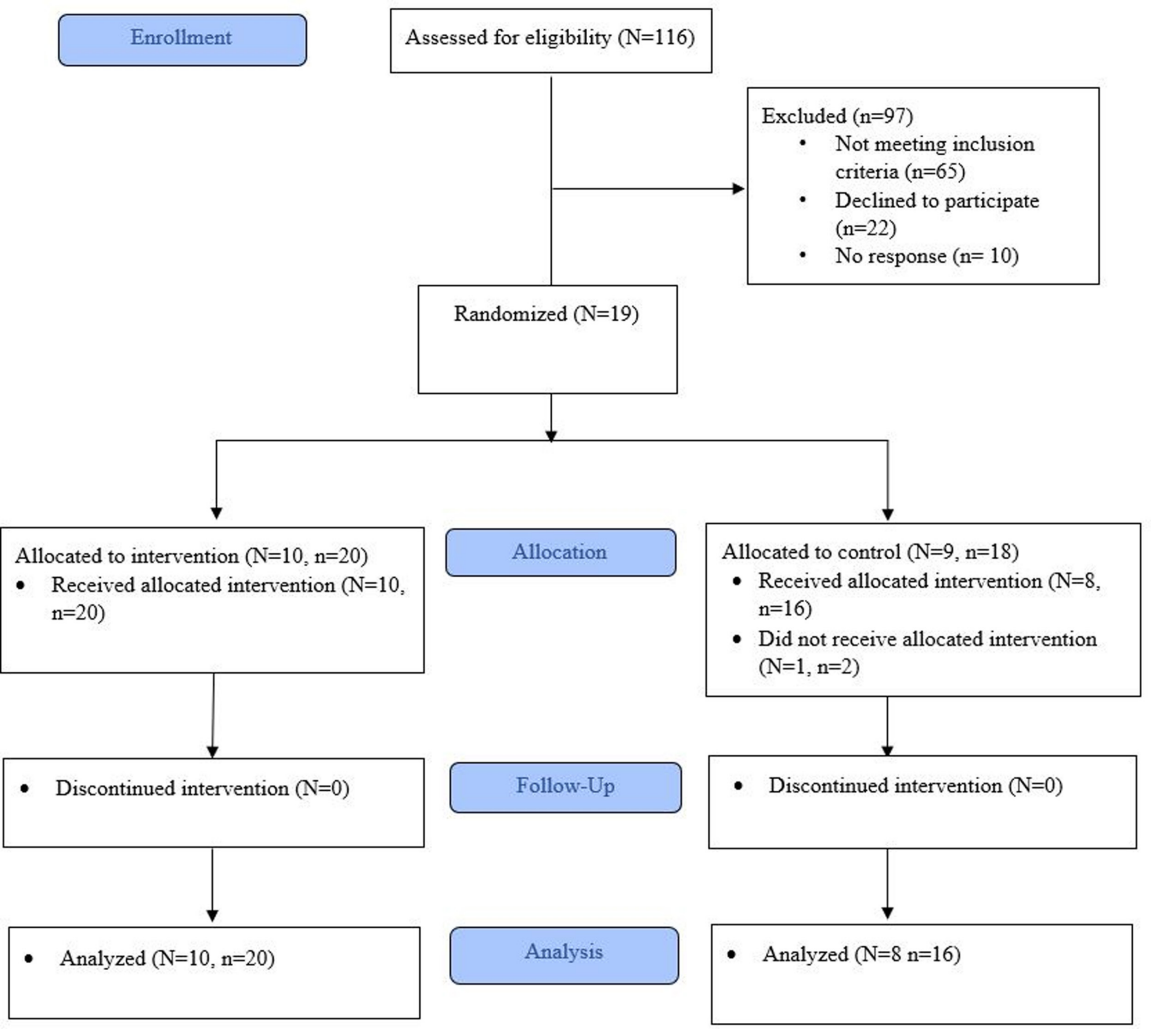
Patient flowchart. N, number of patients; n, number of lower extremities.

Figure 2 illustrates the Consort flow diagram, outlining the recruitment and participation of study participants. Nineteen individuals initially met the inclusion and exclusion criteria; however, one participant withdrew from the study before baseline assessment due to time constraints. This led to a total of 18 participants (Age: IG = 51.10 ± 9.86 years, CG = 52.38 ± 7.44 years, *p* = 0.760; persistency of symptoms after clearance of acute infection: IG = 295.60 ± 224.92 days, CG = 304.50 ± 179.45 days, *p* = 1.0) including *n* = 20 LE in the IG and *n* = 16 LE in CG. Baseline clinical characteristics revealed that the IG had a higher incidence of pneumonia during COVID‐19 acute infection (*p* = 0.043), and higher levels of oxygen at home (*p* = 0.040) than the CG. Additionally, the IG had lower BMI (*p* = 0.016) and poorer cognitive function (*p* = 0.014), while other characteristics did not exhibit significant differences between groups (Table 1).

**TABLE 1:** Demographics and clinical characteristics.

	Intervention (*N* = 10)	Control (*N* = 8)	*p*‐Value (effect size)
Demographics, *n* (%) or mean ± SD			
Age (years)	51.10 ± 9.86	52.38 ± 7.44	0.760 (0.3)
Sex (Female)	7 (70)	6 (75)	0.814 (0.11)
BMI (kg/m^2^)	30.28 ± 5.2	37.03 ± 5.35	0.016 (1.28)
Ethnicity (Non‐Hispanic)	7 (70)	7 (87.5)	0.670 (0.61)
Clinical characteristics, *n* (%) or mean ± SD			
Diabetes	3 (30)	3 (37.5)	0.737 (0.15)
Hypertension	5 (50)	3 (37.5)	0.596 (0.28)
Hyperlipidemia	2 (20)	3 (37.5)	0.410 (0.39)
Prev. cancer	2 (20)	0	0.180 (0.66)
Pneumonia during COVID‐19	4 (40	0	0.043 (1.08)
Current shortness of breath	9 (90)	8 (100)	‐
Respiratory rehabilitation	2 (20)	3 (37.5)	0.410 (0.39)
Walking aid	4 (40)	1 (12.5)	0.236 (0.87)
Days of hospitalization (days)	28.10 ± 27.82	7.75 ± 6.23	0.061 (0.95)
Supplemental oxygen during hospitalization	7 (70)	5 (62.5)	0.737 (0.11)
ICU admission	6 (60)	3 (37.5)	0.343 (0.45)
Persistency of symptoms after acute infection (days)	295.60 ± 224.92	304.50 ± 179.45	1.000 (0)
Oxygen at home	6 (60)	1 (12.5)	0.040 (1.1)
Patient‐reported outcomes, mean ± SD score			
Sleep quality (PSQI)	11.40 ± 1.22	11.00 ± 1.25	0.624 (0.1)
Independence in Daily Activities (ADL)	5.27 ± 0.51	5.52 ± 0.29	0.300 (0.2)
Independence in Instrumental Activities (IADL)	6.32 ± 0.7	7.00 ± 0.49	0.054 (0.37)
Cognitive function (MoCA)	24.44 ± 1.01	26.37 ± 0.94	0.014 (0.66)
Mobility/Tiredness	4.50 ± 0.29	4.40 ± 0.29	0.651 (0.11)
Pain (VAS)	5.44 ± 1	3.94 ± 1.02	0.053 (0.49)
Depression (CES‐D)	19.90 ± 3.37	20.30 ± 3.43	0.581 (0.03)
Anxiety (BAI)	21.80 ± 3.08	21.80 ± 3.2	1.000 (0)
Quality of Life (PROMIS)	26.50 ± 1.75	28.70 ± 1.97	0.154 (0.39)
Lower extremity characteristics, *n* (%) or mean ± SD			
Fatigue	9 (90)	8 (100)	0.357 (0.44)
Weakness	9 (90)	7 (87.5)	0.867 (0.07)
Muscle pain	7 (70)	8 (100)	0.090 (0.87)
Atrophy	5 (50)	5 (62.5)	0.596 (0.25)
Numbness	6 (60)	5 (62.5)	0.914 (0.05)
GNMe (iEMG)	357.06 ± 11.77	362.87 ± 8.87	0.095 (0.6)
Plantar OxyHb (%)	0.56 ± 0.08	0.61 ± 0.1	0.103 (0.56)

*Note*: Variables are expressed as means ± standard deviation.

Abbreviation: ADL, Katz Index of independence in activities of daily living; BAI, Beck Anxiety Index; BMI, Body mass index; CES‐D, Center for Epidemiologic Studies Depression Scale; GNMe, gastrocnemius muscle endurance; IADL, Lawton–Brody Instrumental Activities of Daily Living Scale; ICU, intensive care unit; iEMG, integrated electromyography unit; MoCA, Montreal Cognitive Assessment; OxyHb, Oxyhemoglobin; Prev., previous; PROMIS, Patient‐Reported Outcomes Measurement Information System; PSQI, Pittsburg sleep questionnaire index; VAS, Pain Visual Analog Scale.

### 3.2 Muscle perfusion outcomes

**FIGURE 3:**
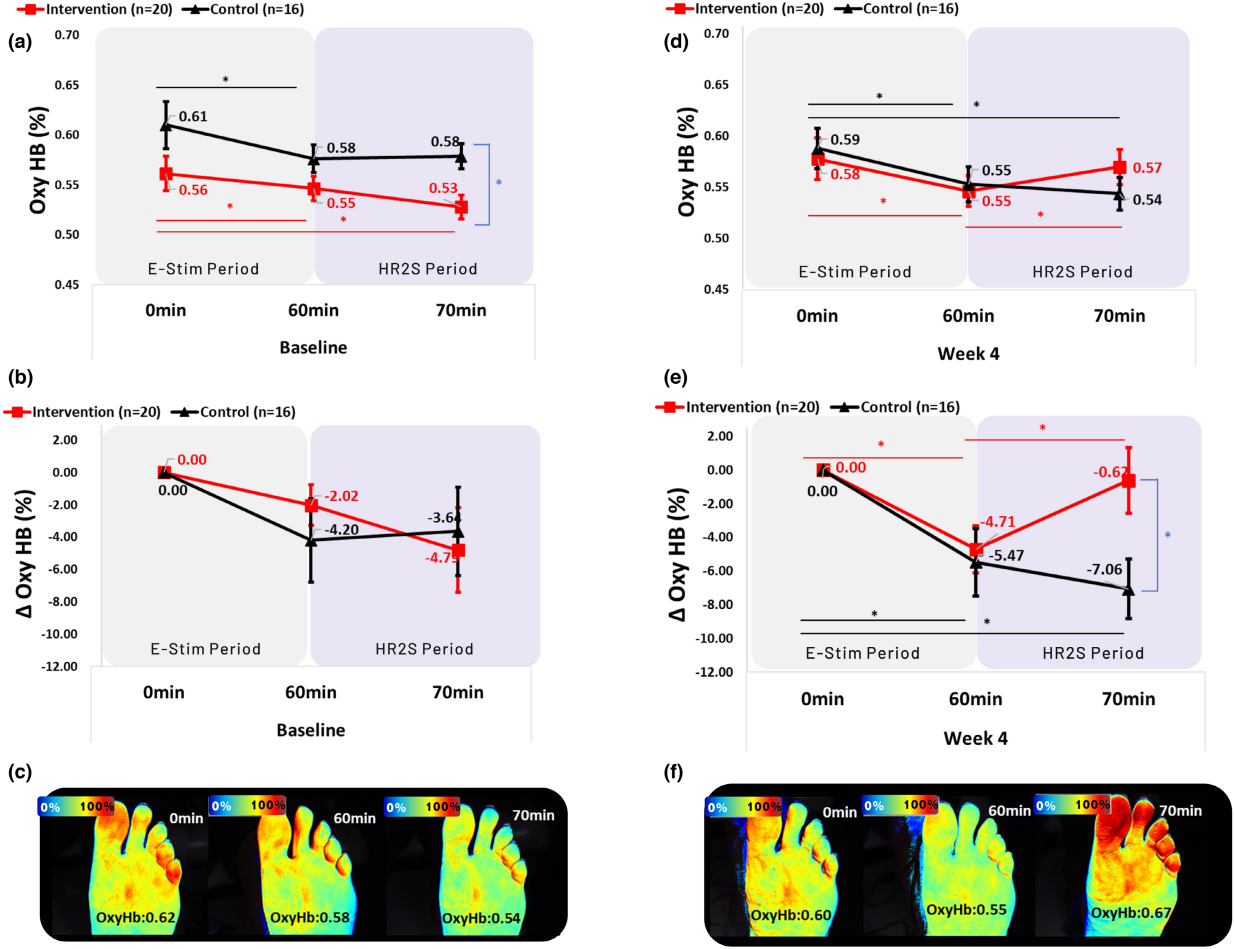
Oxyhemoglobin comparison at baseline and 4 weeks within and between groups. Oxyhemoglobin, OxyHb; E‐Stim, electrical stimulation; HR2S, hemodynamic response to stress; min, minutes; Δ, Delta. Generalized Estimating Equations were performed to assess the group × time × effect interaction of E‐Stim over OxyHb at 0, 60, and 70 min within and between groups. Baseline (a) Absolute and (b) Normalized to 0% change ΔOxyHb values (e.g., [OxyHb at *t*_0_ − OxyHb at *t*_60_ or *t*_70_]/[OxyHb at *t*_0_] * 100) in each time point. (c) A typical case of a patient in the intervention group showing a continuous decrease of OxyHb values after stopping 1 h E‐Stim for 10 min (70 min). Four weeks (d) Absolute and (e) Normalized to 0% change ΔOxyHb values (e.g., [OxyHb at *t*_0_ − OxyHb at *t*_60_ or *t*_70_]/[OxyHb at *t*_0_] * 100) in each time point. (f) A typical case of a patient from the intervention group showing a regain of OxyHb values after stopping 1 h E‐Stim for 10 min (70 min). * Statistically significant (*p* ≤ 0.05).

At baseline, both groups showed a decrease in OxyHb between *t*_0_ and *t*_60_ (IG: 0.56 ± 0.02% vs. 0.55 ± 0.01%, *p* = 0.046, *d* = 0.145; CG: 0.61 ± 0.02% vs. 0.58 ± 0.01%, *p* = 0.026, *d* = 0.490) and between *t*_0_ and *t*_70_ (IG: 0.53 ± 0.01%, *p* = 0.021, *d* = 0.520; CG: 0.58 ± 0.01%, *p* = 0.060, *d* = 0.423, Figure 3a). The IG showed lower OxyHb at *t*_70_ (*p* = 0.004, *d* = 1.204) compared to the CG. Group × time × effect interaction was not significant between groups (*p* = 0.179, Wald Chi‐square = 3.436). Normalized OxyHb values showed a similar but nonsignificant decline between *t*_0_ and *t*_60_ (IG: −2.02 ± 1.27%, *p* = 0.113, *d* = 0.516; CG: −4.20 ± 2.58%, *p* = 0.103, *d* = 0.594) and between *t*_0_ and *t*_70_ (IG: −4.79 ± 2.62%, *p* = 0.067, *d* = 0.593; CG: −3.64 ± 2.72%, *p* = 0.181, *d* = 0.489) in both groups (Figure 3b **).** Group × time × effect interaction was not significant between groups (*p* = 0.314, Wald Chi‐square = 2.316) for normalized OxyHb values at baseline.

After 4 weeks of intervention, both groups showed a decrease in OxyHb between *t*_0_ and *t*_60_ (IG: 0.58 ± 0.02% vs. 0.55 ± 0.02%, *p* < 0.001, *d* = 0.402; CG: 0.59 ± 0.02% vs. 0.55 ± 0.02%, *p* = 0.003, *d* = 0.488). However, at *t*_70_, the IG showed a significant increase in OxyHb compared to *t*_*60*_ (0.57 ± 0.02%, *p* = 0.040, *d* = 0.334), contrary to the CG, which continued to decline (0.54 ± 0.02%, *p* < 0.001, *d* = 0.632, Figure 3d). Group × time × effect interaction was significant between groups (*p* = 0.022, Wald Chi‐square = 7.639). Normalized OxyHb values in both groups showed a decrease in OxyHb between *t*_0_ and *t*_60_ (IG: −4.71 ± 1.39%, *p* < 0.001, *d* = 1.099; CG: −5.47 ± 2.01%, *p* = 0.006, *d* = 0.993). However, at *t*_70_, the IG showed a significant increase in OxyHb compared to *t*_60_ (0.62 ± 1.93%, *p* = 0.037, *d* = 0.558), contrary to the CG, which continued to decline (−7.06 ± 1.78%, *p* < 0.001, *d* = 1.448, Figure 3e). The IG showed higher OxyHb at *t*_70_ (*p* = 0.004, *d* = 0.828) compared to the CG. Group × time × effect interaction was significant between groups (*p* = 0.022, Wald Chi‐square = 7.592) for normalized values at 4 weeks. Similar results were seen for muscle perfusion adjusted to potential confounders (Table S1).

### 3.3 Muscle endurance outcomes

**FIGURE 4:**
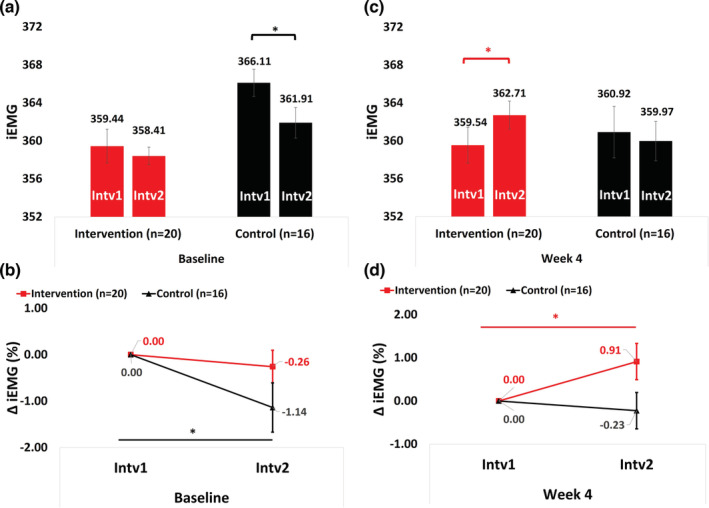
Gastrocnemius muscle endurance at baseline and 4 weeks within and between groups. iEMG, integrated surface electromyogram unit; Intv_1_, Interval 1 (0–5 min); Intv_2_, Interval 2 (55–60 min); Δ: delta. Generalized Estimating Equations were performed to assess the group × time × effect interaction of E‐Stim over GNMe (i.e., Intv_1_ and Intv_2_) within and between groups. Baseline (a) Absolute and (b) Normalized to 0% change Δ GNMe values ([GNMe at Intrv_2_ − GNMe at Intrv_1_]/[GNMe at Intrv_1_] * 100) in each time point. Four weeks (c) Absolute and (d) Normalized to 0% change Δ GNMe values ([GNMe at Intrv_2_ − GNMe at Intrv_1_]/[GNMe at Intrv_1_] * 100) in each time point. * Statistically significant (*p* ≤ 0.05).

At baseline, neither group showed improvement in GNMe. The IG's GNMe did not change between Intv_1_ and Intv_2_ (360.84 ± 1.79 vs. 359.81 ± 0.97, *p* = 0.413, *d* = 0.164), while the CG showed a decline (364.36 ± 1.41 vs. 360.16 ± 1.78, *p* = 0.030, *d* = 0.675, Figure 4a). Group × time × effect interaction was not significantly different (*p* = 0.171, Wald Chi‐square = 1.871). Similar declining trends were observed for normalized GNMe values between Intv_1_ and Intv_2_ in the IG (−0.26 ± 0.35%, *p* = 0.465, *d* = 0.241) and the CG (−1.14 ± 0.53%, *p* = 0.032, *d* = 0.785, Figure 4b). No significant group × time × effect interaction was found for normalized GNMe values at baseline (*p* = 0.167, Wald Chi‐square = 1.909).

After 4 weeks of intervention, the IG exhibited a significant increase in GNMe between Intv_1_ and Intv_2_ (359.88 ± 2.06 vs. 363.04 ± 1.56, *p* = 0.031, *d* = 0.397), while no significant changes were observed in the CG (360.50 ± 2.74, vs. 359.55 ± 2.04, *p* = 0.522, *d* = 0.102, Figure 4c). A significant group × time × effect interaction was found (*p* = 0.048, Wald Chi‐square = 3.893). Normalized GNMe values also increased between Intv_1_ and Intv_2_ in the IG (0.91 ± 0.42%, *p* = 0.029, *d* = 0.703), whereas no significant changes were observed in the CG (−0.23 ± 0.42%, *p* = 0.592, *d* = 0.200, Figure 4d). The IG exhibited a higher trend than the CG in GNMe at Intv_2_ (*p* = 0.055, *d* = 0.654). There was a trend for a group × time × effect interaction (*p* = 0.055, Wald Chi‐square = 3.674) for normalized GNMe values at 4 weeks. Similar results were observed for muscle endurance adjusted to potential confounders (Table S2).

### 3.4 Association of distal lower extremity perfusion and GNM endurance

**FIGURE 5:**
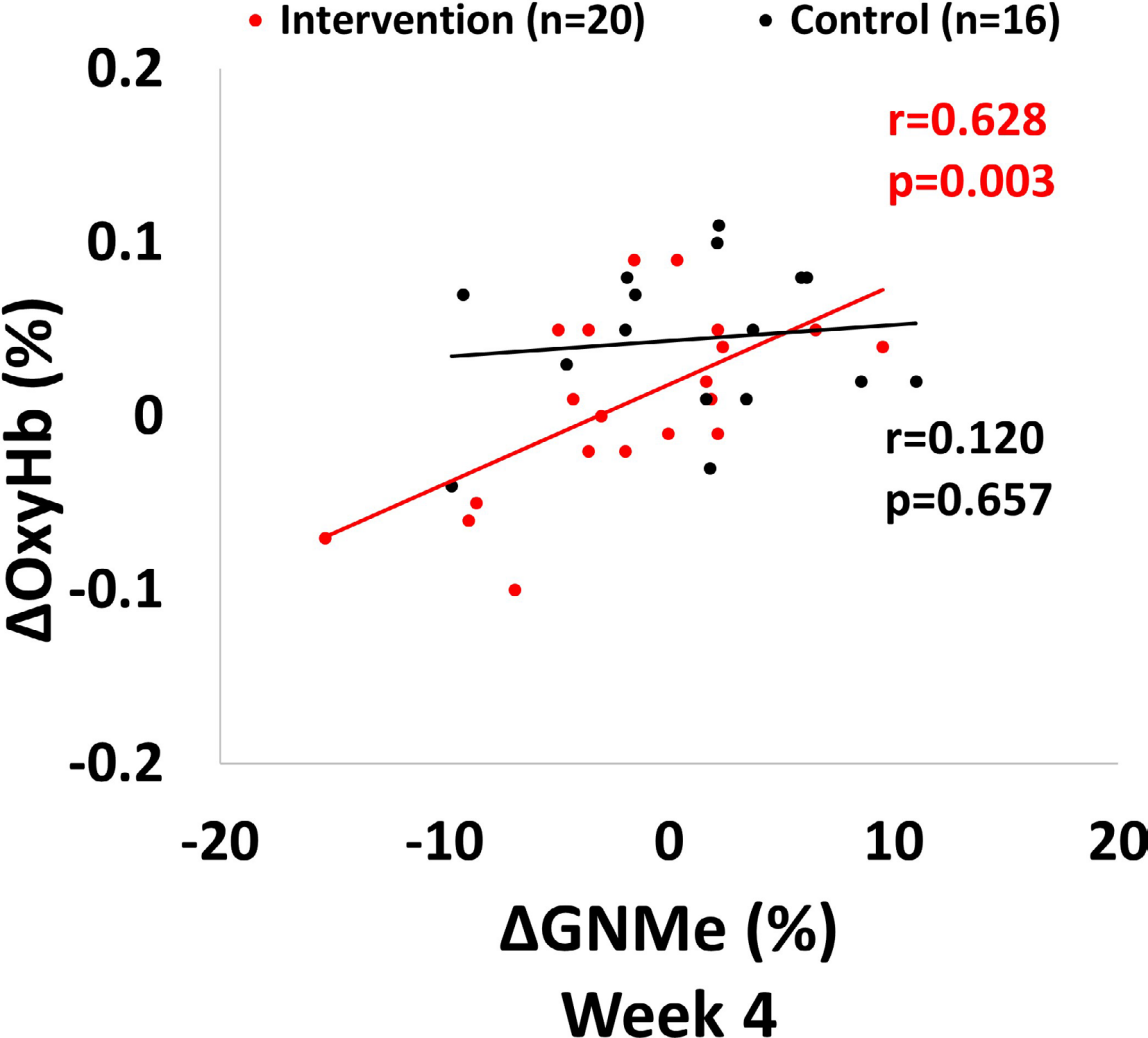
Four weeks correlation comparison between ΔOxyHb and ΔGNMe in both groups. Δ, Delta; OxyHb, Oxyhemoglobin; GNMe, gastrocnemius muscle endurance; ΔGNMe was calculated as GNMe at Intrv_2_ (55–60 min) − GNMe at Intrv_1_ (0–5 min); and ΔOxyHb was calculated as OxyHb at *t*_70_ − OxyHb at *t*_0_. The association between ΔGNMe and ΔOxyHb was explored with Pearson's correlation analysis.

After 4 weeks of E‐Stim therapy, a significant correlation was observed between ΔOxyHb and ΔGNMe (*r* = 0.628, *p* = 0.003) in the IG. Such correlation was not observed in the CG (*r* = 0.120, *p* = 0.657, Figure 5).

### 3.5 Safety, feasibility, and acceptability

After 4 weeks of E‐Stim therapy, both groups showed no change in SatO_2_ values in response to 1 h E‐Stim (IG, *t*_0_: 97.6% vs. *t*_60_: 97.6%, *p* = 0.7; CG: *t*_0_: 97.8% vs. *t*_60_: 97.14%, *p* = 0.33, *d* = 0.34). There was a 100% protocol delivery (no dropouts), accrual recruitment of 4–5 patients/month, 100% outcome measuring (no missed baseline or 4 week visits), and 0% device‐related adverse events. Both groups scored an average 92.8% on ease of therapy self‐administration (strongly agreed on ease of use for pads placement and device operation).

## 4 DISCUSSION

This study investigated the efficacy of daily self‐administered E‐Stim in promoting LE muscle recovery and improving muscle perfusion and endurance in individuals with LE musculoskeletal PASC. This study utilized NIRS and sEMG assessments and found that after a 4 week intervention period of daily 1 h E‐Stim sessions, participants demonstrated a significant increase in both muscle perfusion and endurance. The findings of this study suggest that self‐administered E‐Stim is a safe and effective therapeutic option for individuals with LE musculoskeletal PASC seeking to improve muscle recovery.

During dynamic muscle‐stress (e.g., exercise), NIRS signals from the muscle tissue reflect myoglobin (Mb) (Bendahan et al., 2017; Davis & Barstow, 2013), a globular protein that stores oxygen intracellularly in the muscles (Meyer, 2004). Consequently, a decrease in muscle oxygen levels (i.e., OxyHb) indicates an increase in intracellular oxygen consumption (Van Beekvelt et al., 2001). In this study, we investigated changes in OxyHb by inducing 1 h of submaximal contraction (E‐Stim) to the GNM. The baseline assessment showed a significant drop in OxyHb in response to 1 h E‐Stim in both groups, indicating an increase in oxygen consumption due to continuous muscle activation.

In healthy individuals, muscle tissue typically shows an immediate recovery of oxygen levels after cessation of exercise‐induced stress (healthy HR2S) (Barron et al., 1997; Meixner et al., 2022; Meneses et al., 2020). This phenomenon, known as excess postexercise oxygen consumption (Børsheim & Bahr, 2003), represents the muscle's attempt to repay the oxygen “debt” incurred during prolonged contractions (Barron et al., 1997; Shang et al., 2013). Cettolo et al. (2007) observed that this recovery is slower in people with sedentary lifestyles. Notably, our study found that individuals with musculoskeletal PASC who had been hospitalized failed to show any such recovery, as none of the participants demonstrated an increase in OxyHb toward basal levels (Impaired HR2S at *t*_70_, Figure 3a).

One important consideration is the heterogeneous composition of slow‐ and fast‐twitch fibers in the GNM, which have different oxygen metabolizing capabilities dependent on their levels of Mb and mitochondria (Edgerton et al., 1975). Slow‐twitch fibers have higher levels of both components, resulting in a larger NIRS signal capture (Jansson & Sylvén, 1983). Conversely, fast‐twitch fibers produce greater force but are quicker to fatigue and require longer recovery times (Lievens et al., 1985). When fast‐twitch fibers are pushed beyond their failure point, slow‐twitch fibers take over to continue muscle contraction, indicating that after 1 h of E‐Stim, the majority of the OxyHb NIRS signal after 10 min from stopping E‐Stim therapy (*t*_60_–*t*_70_) represents Mb and mitochondrial recovery (Schmitz, 2013). In COVID‐19 patients with a history of severe illness, there is myofibrillar breakdown related to mitochondrial autophagy (Piotrowicz et al., 2021). Baratto et al. suggested that this could lead to impaired muscle oxygen extraction (Baratto et al., 2021), which may be one reason for the observed baseline dysfunctional muscle HR2S in all participants of this study.

The evidence on post‐COVID‐19 exercise‐induced hypoxia in previously hospitalized patients is compelling. A recent cohort study of 26 hospitalized patients found a 50% incidence of hypoxia during a 6 min walking test prior to discharge (Fuglebjerg et al., 2020). Other randomized studies on post‐COVID‐19 patients who underwent mild exercise showed impaired systemic oxygen extraction (Singh et al., 2022), and peripheral muscle oxygen extraction compared to controls (Baratto et al., 2021). Longobardi et al. (2022) suggested that peripheral metabolic factors affected by COVID‐19 may impair the rate at which oxygen uptake adjusts to changes in energy. While it is unclear whether this impairment is related to mitochondrial dysfunction, some studies suggest that prolonged periods of muscle inactivity, such as those experienced during hospitalization or bed rest, can worsen mitochondrial conditions (Faist et al., 2001; Powers et al., 2012). Taken together, these findings suggest that exercise may induce hypoxia in post‐COVID‐19 patients previously hospitalized, leading to rapid tissue oxygen desaturation.

To investigate the possible role of peripheral oxygen as a marker of muscle perfusion impairment or improvement in individuals with musculoskeletal PASC, we reexamined HR2S at 4 weeks for both groups. Consistent with previous findings (Hansen et al., 2000), both the IG and CG showed a similar drop in OxyHb at *t*_60_. However, when the E‐Stim was stopped for 10 min, only the IG showed a recovery in OxyHb (Figure 3d,e). It has been previously reported that muscle activity can stimulate mitochondrial respiration (Tonkonogi et al., 1998), and enhanced mitochondrial capacity has been linked to endurance training, whether physical (Daussin et al., 2008; Porter et al., 2015) or through E‐Stim therapy (Daussin et al., 2008; Porter et al., 2015). Therefore, we speculate that the 4 week continuous muscle activation induced by E‐Stim therapy might have enhanced the mitochondrial recovery of gastrocnemius myocytes in the IG, leading to a reperfusion reaction similar to that observed in healthy subjects (Barron et al., 1997; Meneses et al., 2020; Shang et al., 2013). However, further studies are necessary to confirm this speculation.

An additional objective measure to assess the improvement in muscle endurance is through iEMG analysis (Zulbaran‐Rojas et al., 2022), which reflects increased muscle fiber activation (Cettolo et al., 2007). In our overall cohort, at baseline, we observed a decline in muscle endurance during the 1 h E‐Stim session (Figure 4a), which was expected given the impaired oxygen metabolism seen in PASC patients, leading to faster muscle fatigue (Nosaka et al., 2011). Previous studies have reported that muscle fatigue can be reduced after 2–4 weeks of E‐Stim therapy as a result of muscle adaptation to induced muscle damage (Clarkson et al., 1992; McHugh, 2003). Consistent with these findings, our study demonstrated that the intervention group had increased muscle endurance after 4 weeks of E‐Stim, in response to continuous muscle contraction. This suggests that only the intervention group regained muscle endurance as a response to E‐Stim therapy. Our results are also in line with our previous study, which reported improved muscle endurance in response to lower extremity E‐Stim therapy (Zulbaran‐Rojas et al., 2022).

Recent studies suggest that endurance training may lead to an increase in capillary density (Hirai et al., 2015; Hudlická et al., 1982; McGuire & Secomb, 2003) and angiogenesis in the LE in as little as 4 weeks (Hoier et al., 2012). However, post‐COVID rehabilitation guidelines recommend that patients with PASC limit their physical activity, making recovery of muscle deterioration challenging (Barker‐Davies et al., 2020). Fortunately, recent reviews suggest that E‐Stim therapy can improve muscle endurance (Nussbaum et al., 2017) and perfusion (Burgess et al., 2021), similar to light‐intensity exercise. In preclinical studies, E‐Stim has also been shown to induce angiogenesis in as little as 2 days (Clemente & Barron, 1993). Our study found that the IG demonstrated an association (Figure 5b) between an increase in GNM endurance in response to 1 h E‐Stim (Δ0‐60 min) and a greater recovery of OxyHb (Δ0‐70 min) after 4 weeks of therapy. This suggests that an increase in GNM endurance can lead to a higher recovery of muscle perfusion. Therefore, E‐Stim may be an effective therapy for improving muscle endurance and recovering a healthy HR2S (Hendrickse & Degens, 2019). However, as the study did not assess tissue samples or biopsies, the mechanism underlying this effect, such as angiogenesis, cannot be confirmed.

While this study used NIRS imaging of the plantar foot muscles rather than the gastrocnemius, it is important to note that this design does not necessarily introduce a confounding factor. Many exercises that involve the calf muscle, such as running, cycling, and calf raises, also involve muscular contribution from the foot muscles. However, since E‐Stim precludes muscular contributions outside the gastrocnemius, it is reasonable to assume that most changes observed pre‐ versus post‐E‐Stim therapy are attributable to the gastrocnemius itself. Nonetheless, it should be acknowledged that a limitation of this study is the lack of NIRS imaging specifically targeting the gastrocnemius muscle.

### 4.1 Study limitations

This study has some limitations that should be considered when interpreting the results. First, the sample size may not be large enough to confirm all observations, and future larger studies are warranted to examine potential differences among COVID‐19 variants or measure specific indicators of muscle damage. Second, functional outcomes were not assessed, and exercise‐induced hypoxia was not measured with cardiopulmonary exercise testing. Additionally, future studies could directly measure OxyHb from the GNM or assess other LE muscles in addition to the GNM. Third, physiologic changes were based on clinical observations, and histologic studies are needed to test angiogenesis or intracellular changes. Fourth, three patients in the CG recognized they had a sham device during the study, but they were not unblinded. Fifth, adherence to therapy and compliance were monitored by weekly phone calls, but no objective or device‐tracking method was used. Finally, baseline parameters were significantly different in pneumonia during acute infection, and oxygen at home, suggesting that the IG was more ill than the CG. Despite these limitations, we observed medium to large effect sizes for the benefit of E‐Stim, which was safe, easy to administer, and highly acceptable. Future efforts are needed to confirm or refute the initial compelling findings of this study.

### 4.2 Interpretation

Our study investigated the safety and potential benefits of a 4 week self‐administered E‐Stim therapy program in individuals with musculoskeletal PASC LE symptoms who were previously hospitalized. We found that a daily 1 h session of E‐Stim was both safe and well‐tolerated and may lead to improved muscle perfusion and endurance. Furthermore, we observed a potential benefit for GNM vascular improvement leading to a healthier HR2S. These findings suggest that E‐Stim therapy is a practical and promising intervention for individuals with musculoskeletal PASC LE symptoms seeking to improve their functional recovery.

## AUTHOR CONTRIBUTIONS

BN and AZ conceived and designed research; AZ, RB, and AF performed experiments; ML analyzed data; BN, AZ, AF, MGF, and GS interpreted results of experiments; AZ, ML, MGF, and AB prepared figures; AZ, RB, AF, ML, and GS drafted the article; BN, MGF, FS, AB, and DM edited and revised the article; AZ, ML, RB, AF, MGF, SG, AB, DM, FS, and BN approved final version of the article.

## FUNDING INFORMATION

This study was supported in part by Avazzia Inc. (DL, TX, US), which is the manufacturer of the Tennant Biomodulator®. The sponsor did not have any decision or contribution to the review, approval, and submission of this article.

## CONFLICT OF INTEREST STATEMENT

No conflicts of interest, financial or otherwise, are declared by the authors.

## ETHICS STATEMENT

This study was approved by the local Institutional Review Board (IRB #H‐47781) at Baylor College of Medicine. All participants signed the informed consent form before study enrollment. The study was registered on ClinicalTrials.gov (Identifier: NCT05198466) and followed the Consolidated Standards of Reporting Trials (CONSORT) guidelines for randomized clinical trials. The study was conducted in compliance with the Declaration of Helsinki.

## Supporting information


Table S1.
Supplemental Table 2.
