# Supplementary material for: Electrical stimulation to regain lower extremity muscle perfusion and endurance in patients with post‐acute sequelae of SARS CoV‐2: A randomized controlled trial
Source: Physiol Rep. 2023 Mar 10;11(5):e15636. doi: 10.14814/phy2.15636 (PMC10006649; doi:10.14814/phy2.15636)
Supplement: Supplementary file 1 — Table S1. Supplemental Table 2. [file PHY2-11-e15636-s001.docx]

**Supplemental table 1.** Muscle perfusion outcomes adjusted to potential confounders.

|  | **Control (n=16)** | | | | | | **Intervention (n=20)** | | | | | |  |  |
| --- | --- | --- | --- | --- | --- | --- | --- | --- | --- | --- | --- | --- | --- | --- |
|  |  |  |  | Time×effect  P-value  (Cohen's-d) | | |  |  |  | Time×effect  P-value  (Cohen's-d) | | | Group×effect  P-value (Cohen's-d) | Group×time  effect:  P-value  (Wald Chi-square) |
|  | *t_0_* | *t_60_* | *t_70_* | *t_0_ vs t_60_* | *t_0_ vs t_70_* | *t_60_ vs t_70_* | *t_0_* | *t_60_* | *t_70_* | *t_0_ vs t_60_* | *t_0_ vs t_70_* | *t_60_ vs t_70_* | *t_70_* |  |
| **Baseline** |  |  |  |  |  |  |  |  |  |  |  |  |  |  |
| OxyHb (%) | 0.64 ± 0.03 | 0.60 ± 0.02 | 0.61 ± 0.02 | 0.014  (0.405) | 0.041  (0.304) | 0.762  (0.129) | 0.54 ± 0.03 | 0.53 ± 0.02 | 0.51 ± 0.02 | 0.043  (0.090) | 0.019  (0.270) | 0.084  (0.229) | 0.006 | 0.143  [3.892] |
| Normalized to 0% OxyHb, (%) | 0 | -4.20 ± 2.58 | -3.64 ± 2.72 | 0.103  (0.594) | 0.181  (0.489) | 0.697  (0.055) | 0 | -2.02 ± 1.27 | -4.80 ± 2.62 | 0.113  (0.516) | 0.067  (0.594) | 0.176  (0.760) | 0.760 | 0.314  [2.320] |
| **4-weeks** |  |  |  |  |  |  |  |  |  |  |  |  |  |  |
| OxyHb (%) | 0.60 ± 0.03 | 0.56 ± 0.02 | 0.55 ± 0.02 | 0.004  (0.405) | <0.001  (0.506) | 0.206  (0.129) | 0.57 ± 0.02 | 0.54 ± 0.02 | 0.56 ± 0.02 | <0.001  (0.344) | 0.478  (0.115) | 0.041  (0.282) | 0.758 | 0.021  [7.720] |
| Normalized to 0% OxyHb (%) | 0 | -5.47 ± 2.01 | -7.06 ± 1.78 | 0.006  (0.994) | <0.001  (1.448) | 0.082  (0.216) | 0 | -4.71 ± 1.39 | -0.62 ± 1.93 | <0.001  (1.099) | 0.750  (0.104) | 0.037  (0.558) | 0.014 | 0.022  [7.591] |

Mean ± standard error. Body mass index was adjusted as a covariance. OxyHb, Oxyhemoglobin; *t_0_*, 0 minutes; *t_60_*, 60 minutes; *t_70_*, 70 minutes.

**Supplemental Table 2.** Gastrocnemius muscle endurance outcomes adjusted to potential confounders.

|  | **Control (n=16)** | | | **Intervention (n=20)** | | |  |  |
| --- | --- | --- | --- | --- | --- | --- | --- | --- |
|  | Interval1:  0 - 5 min | Interval2: 55 - 60 min | Tim×effect: P-value  (Cohen's-d) | Interval1:  0 - 5 min | Interval2: 55 - 60 min | Time×effect: P-value (Cohen's-d) | Group×effect: Interval2 P-value  (Cohen's-d) | Group×time  effect:  P-value  [Wald Chi-squire] |
| **Baseline** |  |  |  |  |  |  |  |  |
| GNMe | 366.11 ± 2.74 | 361.91 ± 2.04 | 0.030  (0.449) | 359.44 ± 2.06 | 358.41 ± 1.56 | 0.413  (0.129) | 0.066  (0.479) | 0.171  [1.871] |
| Normalized to 0%  GNMe (%) | 0 | -1.14 ± 0.53 | 0.032  (0.785) | 0 | -0.26 ± 0.35 | 0.465  (0.241) | 0.167  (0.495) | 0.167  [1.909] |
| **4-weeks** |  |  |  |  |  |  |  |  |
| GNMe | 360.92 ± 2.74 | 359.97 ± 2.04 | 0.522  (0.101) | 359.54 ± 2.06 | 362.71 ± 1.56 | 0.031  (0.427) | 0.282  (0.375) | 0.048  [3.893] |
| Normalized to 0%  GNMe (%) | 0 | -0.23 ± 0.42 | 0.592  (0.200) | 0 | 0.91 ± 0.42 | 0.029  (0.703) | 0.055  (0.654) | 0.055  [3.674] |

Mean ± standard error. GNMe: Gastrocnemius muscle endurance. Body mass index was adjusted as a covariance.
